# Differential Effects of Angelicin Analogues on NF-*κ*B Activity and IL-8 Gene Expression in Cystic Fibrosis IB3-1 Cells

**DOI:** 10.1155/2017/2389487

**Published:** 2017-09-27

**Authors:** Ilaria Lampronti, Maria Giulia Manzione, Gianni Sacchetti, Davide Ferrari, Susanna Spisani, Valentino Bezzerri, Alessia Finotti, Monica Borgatti, Maria Cristina Dechecchi, Giorgia Miolo, Giovanni Marzaro, Giulio Cabrini, Roberto Gambari, Adriana Chilin

**Affiliations:** ^1^Department of Life Sciences and Biotechnology, University of Ferrara, Via Fossato di Mortara 74, Ferrara, Italy; ^2^Department of Medicine, University of Verona, Strada le Grazie 8, Verona, Italy; ^3^Department of Pathology and Diagnostics, Laboratory of Molecular Pathology, University Hospital of Verona, Verona, Italy; ^4^Department of Pharmaceutical and Pharmacological Sciences, University of Padova, Via Marzolo 5, Padova, Italy; ^5^Center of Biotechnology, University of Ferrara, Via Fossato di Mortara 64/b, Ferrara, Italy

## Abstract

The angelicin analogue 4,6,4′-trimethylangelicin (TMA) was recently reported as a strong inhibitor of nuclear factor-*κ*B (NF-*κ*B) activity and of the expression of the interleukin-8 (IL-8) gene in bronchial epithelial cells in which the inflammatory response has been challenged with *P. aeruginosa*, the most common bacterium found in the airways of patients affected by cystic fibrosis (CF). These findings encouraged us to analyze new synthetic analogues of TMA in order to evaluate their biological activities on human bronchial epithelial CF IB3-1 cells and to find more potent anti-NF-*κ*B agents exhibiting only minor antiproliferative effects. Analogues able to inhibit NF-*κ*B/DNA interaction at lower concentration than TMA were found and selected to investigate their biological activity on IB3-1 cells induced with TNF-*α*. In this biological system, NF-*κ*B-mediated IL-8 gene expression was investigated. Some analogues showed similar activity to the lead compound TMA. Other analogues displayed higher activities; in particular, the most interesting compounds showing relevant anti-inflammatory effects were found to cause 56–83% reduction of IL-8 mRNA expression at low concentrations (1–10 *μ*M), without changes in cell proliferation pattern, demonstrating their potential interest for a possible development of anti-inflammatory therapy of cystic fibrosis.

## 1. Introduction

**Figure 1:**
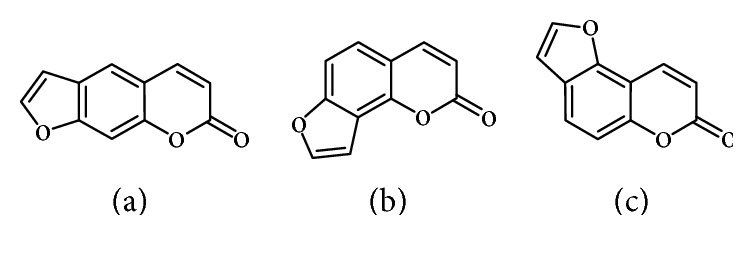
Chemical skeleton of linear and angular furocoumarins.

Psoralens, also known as furocoumarins, are naturally occurring or synthetic tricyclic aromatic compounds, deriving from the condensation of a coumarin nucleus with a furan ring [1, 2]. The furan moiety may be fused in different ways producing several isomers: linear isomers (psoralens; Figure 1(a)) and angular isomers (angelicins, Figure 1(b); allopsolarens, Figure 1(c)) [3]. The most abundant linear furocoumarins are psoralen, xanthotoxin, and bergapten; whereas the angular type is mostly represented by angelicin [3].

Furocoumarins are found in natural plant extracts belonging to angiosperm families, formerly used in popular medicine: for example, angelicin is present in *Angelica archangelica* (Apiaceae), allopsoralen in *Mammea americana* (Guttiferae), and psoralens in *Psoralea corylifolia* (Leguminosae) and *Amni majus* (Apiaceae) [4–6]. Bergapten is found in *Citrus bergamia* (Rutaceae) [7].

At present, several psoralens are used also in conventional medicine in the treatment of various skin diseases (mainly vitiligo and psoriasis) [8], using PUVA therapy (Psoralen plus UVA light) [9]. The natural angular angelicin (ANG), used in folk medicine, and its synthetic derivative 4,6,4′-trimethylangelicin (TMA) were described in recent studies of our research group [10, 11]. TMA was found to be potentially useful in the treatment of cystic fibrosis (CF), thanks to its anti-inflammatory activity and its potentiating action on the CFTR membrane channel whose dysfunction causes that disease. Moreover, TMA resulted to be a new corrector, showing actually a third pharmacological activity [12, 13].

Cystic fibrosis, a genetic disease that primarily affects the lungs and digestive system, is the most common autosomal recessive disease in Caucasians associated with early death [14, 15]; it is a complex multisystem disease caused by defects in a single gene that is CFTR (cystic fibrosis transmembrane conductance regulator), which encodes for a chloride channel expressed in several epithelia [16]. Pulmonary inflammation is responsible for most of the morbidity and mortality because the most important clinical complication is focalized in the airway tract of affected patients [17, 18]. In the healthy lung, CFTR is detectable on the apical membrane of ciliated cells within the gland ducts and in the superficial epithelium of healthy individuals. In CF, the submucosal glands and distal airways are obstructed by thick tenacious secretions, resulting in a failure of normal mucociliary clearance and defective airway defense mechanisms against inflammation and bacterial infection. Therefore, inhibition of the proinflammatory pathways in CF appears to be clinically relevant [19–21].

In this context, the contribution of nuclear factor-kappaB (NF-*κ*B) in chronic inflammatory and autoimmune diseases is well known in CF. The activation of NF-*κ*B triggers proinflammatory cytokine production [22, 23]. Therefore, targeting NF-*κ*B might be a very interesting strategy in CF, since NF-*κ*B has been firmly linked to the IL-8 (interleukin-8) gene expression [24], which is a key chemokine activated in *Pseudomonas aeruginosa* induction of state of CF lung. Targeting NF-*κ*B has been reported using DNA-based drugs, such as decoy oligonucleotides [24–27], as well as low molecular weight molecules [28, 29]. However, NF-*κ*B has also been reported as an antiapoptotic factor related to cell growth, suggesting that NF-*κ*B inhibitors (as found for TMA) might exhibit antiproliferative and/or cytotoxic effects.

In order to improve the anti-inflammatory activity of TMA and to reduce some related cytotoxic effects, we analyzed new thirty-eight TMA derivatives belonging to different chemical classes (series) and reported in Table 1, in order to verify whether compounds are present displaying effects on NF-*κ*B, inflammatory-related proteins without major alteration of cell growth potential.

## 2. Materials and Methods

### 2.1. Synthesis and Characterization of TMA Analogues

TMA (4,6,4′-trimethylangelicin) was synthesized at the Department of Pharmaceutical Sciences of the University of Padova [30], and it was dissolved in a mixture of methanol (MeOH) and 3% of dimethyl sulfoxide (DMSO) to obtain the 10 mM stock solution, stored at −20°C in the dark. The working solutions were then obtained using MeOH. TMA analogues were partly synthesized as previously described [31–40] and partly belong to the collection of the Organic Synthesis Lab (Dept. of Pharmaceutical Sciences, University of Padova). The stock solutions (100 mM) of each compound were prepared in DMSO and were diluted with MeOH to obtain the desired working solutions (10 mM-500 *μ*M), immediately before the *in vitro* experiments and treatment of cell cultures. No effect of the maximum concentrations employed on the vehicles on the test performed (cell growth, gene expression) was observed.

### 2.2. EMSA (Electrophoretic Mobility Shift Assays)

Electrophoretic mobility shift assays were performed using double-stranded ^32^P-labeled oligonucleotides as target DNA. Binding reactions were set up as previously described [19] in binding buffer (10% glycerol, 0.05% NP-40, 10 mM Tris-HCl pH 7.5, 50 mM NaCl, 0.5 mM DTT, and 10 mM MgCl2), in the presence of 0.1 *μ*l/20 *μ*l of NF-*κ*B p50 (50 gsu) (Promega, Madison, WI, USA) and 0.25 ng of labeled oligonucleotide, in a total volume of 20 *μ*l. After 30 min binding at room temperature, samples were electrophoresed at constant voltage (200 V for 30 min) through a low ionic strength (0.25 TBE buffer) (1 TBE/40.089 M Tris-borate, 0.002 M EDTA) on 6% polyacrylamide gels until the tracking dye (bromophenol blue) reached the bottom of a 16 cm slab. Gels were dried and exposed for autoradiography with intensifying screens at 80°C. In these experiments, DNA/protein complexes migrate through the gel with slower efficiency. In studies on the inhibitors of protein/DNA interactions, addition of the reagents was as follows: (i) nuclear factors, (ii) active principles, (iii) binding buffer, and (iv) labeled oligonucleotides mimicking the binding sites for TF to be modulated. The nucleotide sequence of double-stranded target DNA utilized in these experiments was 5′-CGC TGG GGA CTT TCC ACG G-3′ (sense strand, NF-*κ*B). The synthetic oligonucleotides utilized in this study were purchased from Sigma Genosys (Sigma Genosys, Cambs, UK). To calculate the effective concentrations, three different autoradiograms obtained from different time exposures were scanned and compared to control EMSA performed without addition of angelicin analogues (two independent experiments were performed).

### 2.3. Cell Cultures

IB3-1 cells, derived from a CF patient with a ΔF508/W1282X mutant genotype and immortalized with adeno12/SV40, were grown in LHC-8 supplemented with 5% FBS in the absence of gentamycin, at 37°C/5% CO_2_ [12].

### 2.4. Cell Proliferation Assays

Monolayers of 60% confluent IB3-1 cells were seeded in 24- or 12-well plates in LHC-8 medium in the presence of 5% FBS. After 24 h, TMA derivatives were added at serial dilutions and incubated for further 2-3 days. The effects of compounds (TMA derivatives) on the cell proliferation were analyzed as elsewhere described. A first analysis was performed with serial dilutions to obtain 50 *μ*M, 200 *μ*M, and 800 *μ*M solutions. In order to determine IC_50_ values, the following concentrations were used 12.5, 25, 50, 100, 200, and 800 *μ*M. In order to prepare the cells for counting, they were washed with PBS 1X and detached with trypsin/EDTA. Cells were suspended in physiological solution and counted with a Z2 Coulter Counter (Coulter Electronics, Hialeah, FL, USA). The cell number/ml was determined as IC_50_ after 2 days of culture, when untreated cells are in log phase of cell growth.

### 2.5. Quantification of IL-8 mRNA Content

Monolayers of 60% confluent IB3-1 cells were seeded in 6- or 12-well plates in LHC-8 medium in the presence of 5% FBS. After 24 h, pure derivatives were added, 5 h before stimulation with TNF-*α* 80 ng/ml (ORF Genetics, Kopavogur, Iceland), and incubated for a further 24 h. Total RNA was extracted using TRIzol Reagent (Sigma-Aldrich, St. Louis, MO) following the manufacturer's instructions. Reverse transcription (RT) was performed using Reverse Transcription System kit (Promega, Madison, WI): 1 *μ*g of total RNA was reverse transcribed in the presence of 5 mM MgCl_2_, 1X reverse transcription buffer (10 mM Tris-HCl, 50 mM KCl, and 0.1% Triton X-100), 1 mM each dNTPs, 20 U recombinant Rnasin Ribonuclease Inhibitor, 15 U AMV Reverse Transcriptase, and 0.5 *μ*g oligo(dT)15 primers in a total volume of 20 *μ*l for 10 min at 70°C and 60 min at 42°C. The resulting cDNA was quantified by relative quantitative real-time PCR (real-time qPCR). For real-time qPCR, 1 *μ*l of cDNA was used for each SYBR Green reaction to quantify the relative expression of IL-8. Each 25 *μ*l of total reaction volume contained 1 *μ*l of cDNA, 10 pmol of primers, 1 iQTM SYBR Green Supermix (Bio-Rad Laboratories Inc., Hercules, CA). Real-time PCRs were performed for a total of 40 cycles (denaturation, 95°C for 10 s; annealing, 68°C for 30 s for IL-8, 65°C for 30 s for IL-6; elongation, 72°C for 60 s) using an iCycler IQ5 (Bio-Rad Laboratories Inc., Hercules, CA). Primer sequences were as follows: IL-8 forward: 5′-GTG CAG TTT TGC CAA GGA GT-3′ and IL-8 reverse: 5′-TTA TGA ATT CTC AGC CCT CTT CAA AAA CTT CTC-3′. The relative proportions of each amplified template were determined utilizing the threshold cycle (C_T_) value for each performed PCR. The DDCt method was used to compare gene expression data. Each sample was quantified in duplicate. Changes in mRNA expression level were calculated following normalization with the GAPDH calibrator gene (housekeeping gene) and expressed as fold change over untreated samples [24]. Duplicates of negative controls (no template cDNA) were also run with every experimental plate to assess specificity and indicate potential contamination.

### 2.6. Bioplex Analysis

Cytokines, released from cells into tissue culture supernatants, were measured by Bioplex cytokine assay (Bio-Rad Laboratories Inc., Hercules, CA) as suggested by the manufacturer (Luminex technology) [41]. The Bioplex cytokine assay is designed for the multiplexed quantitative measurement of multiple cytokines in a single well using as little as 50 *μ*l of sample. In our experiments, the premixed multiplex beads (27-plex) of the Bioplex human cytokines were used. 50 *μ*l of cytokine standards or samples (supernatants recovered from treated cells and diluted to 2 *μ*g/*μ*l) were incubated with 50 *μ*l of anticytokine conjugated beads in 96-well filter plates for 30 min at room temperature with shaking. Plates were washed by vacuum filtration three times with 100 *μ*l of Bioplex wash buffer, 25 *μ*l of diluted detection antibody were added, and plates were incubated for 30 min at room temperature with shaking. After three filter washes, 50 *μ*l of streptavidin-phycoerythrin were added, and the plates were incubated for 10 min at room temperature with shaking. Finally, plates were washed by vacuum filtration three times, beads were suspended in Bioplex assay buffer, and samples were analyzed on a Bio-Rad 96-well plate reader using the Bioplex Suspension Array System and Bioplex Manager software (Bio-Rad Laboratories Inc., Hercules, CA).

## 3. Results

### 3.1. Effects of TMA Derivatives on the *In Vitro* Proliferation of Cystic Fibrosis IB3-1 Cells

In order to obtain preliminary information on the biological properties of TMA derivatives, their effects on cell growth were examined on IB3-1 cell line, excellent biological model to study new potential anti-inflammatory molecules. The use of this cell line to study the expression of proinflammatory molecules under a variety of stimuli is well established. Monolayers of 60% confluent IB3-1 cells were seeded in 12-well plate in LHC-8 medium with 5% FBS. After 24 h, compounds were added at different concentrations and the cell number/ml was determined after further 48 h of culture. All the obtained IC_50_ values are reported in Table 2.

Several TMA analogues displayed very low antiproliferative effects (>800 *μ*M), such as compounds **1**–**4**, **7**, **9**, **12**–**16**, **18**, **27**–**34**, **36**, and **37**. This lack of antiproliferative activity of these angelicin analogues was confirmed in further experiments, fully sustaining the conclusion that these analogues do not cause 50% inhibition of cell growth even when added at high concentration, being therefore of interest in the identification of possible inhibitors of inflammation-related genes without major toxic effects. TMA exhibited an IC_50_ value of 185 ± 30 *μ*M (data not shown).

### *3.2.In Vitro* Inhibition of NF-*κ*B/DNA Interactions

The effects of TMA derivatives on NF-*κ*B- (nuclear factor-kappaB-) mediated induction of proinflammatory genes was verified first *in vitro* by electrophoretic mobility shift assay (EMSA) with the aim to verify and quantify the possible inhibition of NF-*κ*B/DNA interactions. This assay was performed using purified NF-*κ*B p50 and ^32^P-labeled target NF-*κ*B double-stranded oligonucleotide mimicking the NF-*κ*B consensus sequence present within the IL-8 gene promoter.

**Figure 2:**
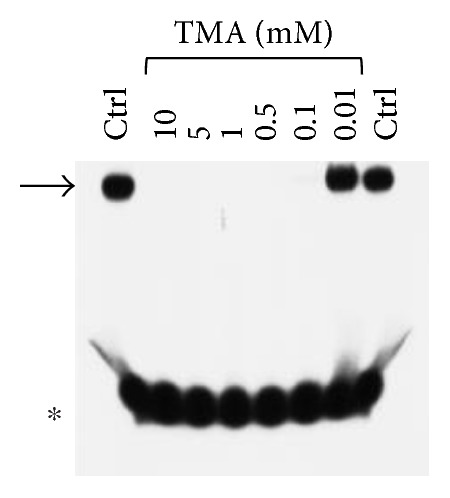
Effects of TMA on the molecular interactions between NF-*κ*B p50 and a ^32^P-labeled target NF-*κ*B double-stranded oligonucleotide. TMA was first incubated with NF-*κ*B, and then the ^32^P-labeled target NF-*κ*B oligonucleotide was added. NF-*κ*B/DNA complexes were analyzed by polyacrylamide gel electrophoresis. Arrow indicates NF-*κ*B/DNA complexes; asterisk indicates the free ^32^P-labeled target NF-*κ*B probe.

We first confirmed that TMA is able to suppress NF-*κ*B/DNA interactions when used at 100 *μ*M concentration (Figure 2); to verify whether new analogues were more active than the TMA lead compound, we performed an EMSA-based screening considering the TMA lowest active concentration to be higher than 20 *μ*M (Figure 2 and data not shown).

**Figure 3:**
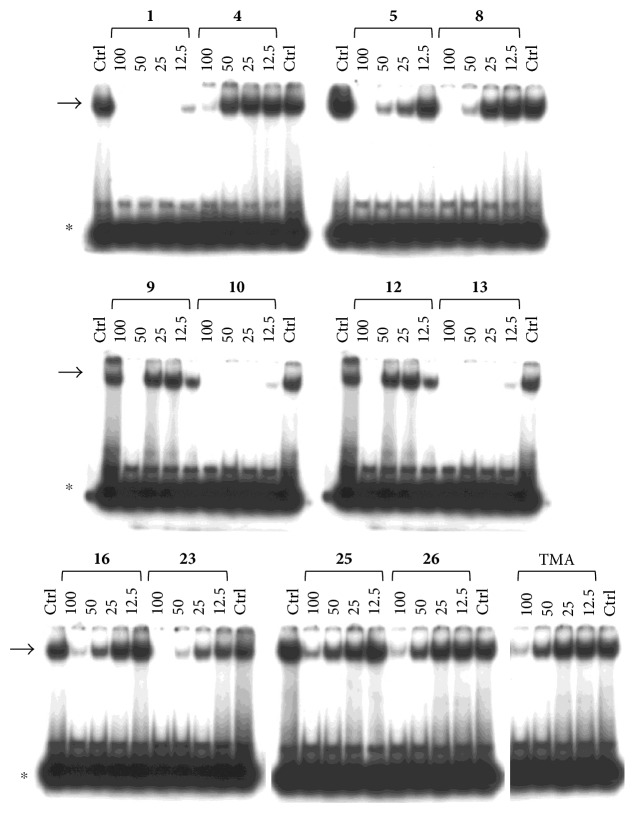
Representative results depicting the effects of TMA analogues (**1**–**26**) at 12.5–100 *μ*M concentrations, compared to TMA in EMSA experiments on the molecular interactions between NF-*κ*B p50 and ^32^P-labeled target NF-*κ*B double-stranded oligonucleotide. Compounds **2**, **3**, **6**, **7**, **11**, **14**, **15**, **17**–**22**, and **24** were not analyzed because the relative EMSA experiments demonstrated low activity (MIC > 100 *μ*M). Arrow indicates NF-*κ*B/DNA complexes; asterisk indicates the free ^32^P-labeled target NF-*κ*B probe.

**Figure 4:**
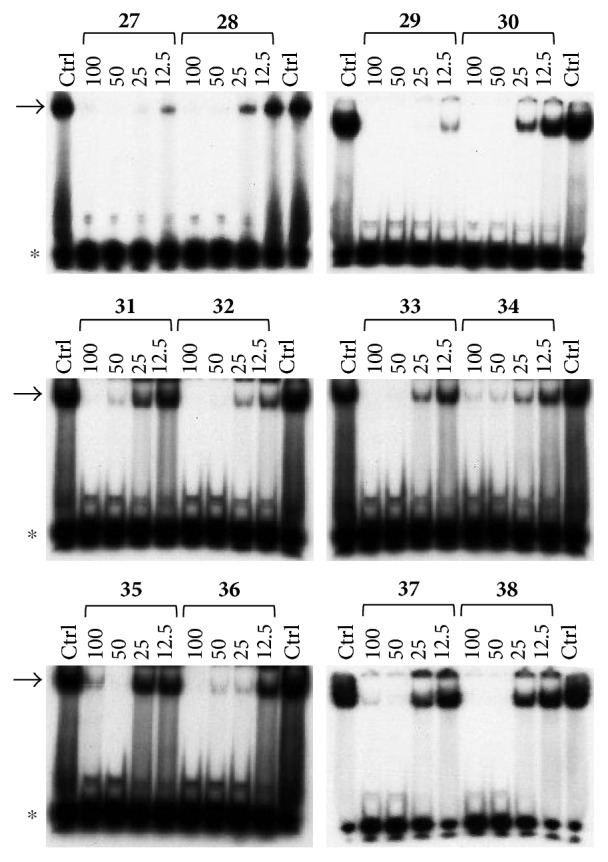
Representative results depicting the effects of TMA analogues (**27**–**38**) at 12.5–100 *μ*M concentrations, compared to TMA in EMSA experiments on the molecular interactions between NF-*κ*B p50 and ^32^P-labeled target NF-*κ*B double-stranded oligonucleotide. Arrow indicates NF-*κ*B/DNA complexes; asterisk indicates the free ^32^P-labeled target NF-*κ*B probe.

Preliminary data demonstrated that, when used at 100 *μ*M concentration, several derivatives fully suppress the interactions between NF-*κ*B p50 and the target DNA, while other molecules (compounds **2**, **3**, **6**, **7**, **11**, **14**, **15**, **17**, **22**, and **24**) were not analyzed because the relative EMSA experiments demonstrated low activity (MIC > 100 *μ*M) (data not shown). According to these first results, further analyses were performed using different dilutions of the selected active compounds, exhibiting MIC values ranging from 12.5 *μ*M to 100 *μ*M, as represented in Figures 3 and 4 and summarized in Table 3, reporting their MIC values.

Among the “4,6,4′ series” (compounds **1**, **2**, and **3**), the most interesting molecule was compound **1**, showing MIC < 12.5 *μ*M, while **4** and **5** derivatives, belonging to the “4,6 series” were active at 75 *μ*M and 25 *μ*M, respectively.

The “6,4′ series,” including **8**, **9**, and **10** analogues, displayed excellent inhibitory activities (35 *μ*M, 12.5 *μ*M, and <12.5 *μ*M MIC, resp.).

In the same figure, we may also observe high activity of **12** and **13** derivatives (“4,4′ series”) showing low MIC value (12.5 *μ*M), while among the “thio-series” (**14**, **15**, and **16**), only **16** demonstrated a 50 *μ*M MIC value. All furoquinolinone analogues, belonging to the “FQ series” (**17**, **18**, and **19**), were considered inactive when compared with TMA (MIC > 100 *μ*M).

Among derivatives belonging to “other angelicins” group, compounds **23**, **25**, and **26** were able to inhibit the NF-*κ*B/DNA interaction showing MIC values of 25–50 *μ*M.

All the derivatives starting from **27** to **38** showed interesting MIC values in EMSA (Figure 4). Indeed, we observed a MIC value < 12.5 *μ*M for the chromone derivative **27**. The 3,4 analogue **28** inhibits the NF-*κ*B/DNA interaction at 15 *μ*M.

Among “difuro series,” we observed a MIC value < 12.5 *μ*M when **29** is added to binding reaction, and we observed an MIC value of 15 *μ*M for derivative **30**.

Triazole analogue **31** demonstrated its activity at 25 *μ*M.

Among “allo series,” two TMA isosters, **32** and **33**, showed MIC values of 12.5 *μ*M and 15 *μ*M, respectively.

Among “pyran series,” compound **36** demonstrated the most interesting inhibitory effect showing 15 *μ*M MIC value; derivative **35** was able to interfere with the NF-*κ*B/DNA interaction at 35 *μ*M MIC. Finally, two coumarin compounds, **37** and **38**, both exhibited active at 25 *μ*M MIC.

In conclusion, several TMA derivatives were found to be more active than the reference compound TMA, such as **1**, **5**, **10**, **12**, **23**, **27**–**29**, **31**, **32**, **34**, and **36**–**38**.

### 3.3. Inhibition of IL-8 mRNA Accumulation in TNF-*α*-Treated IB3-1 Cystic Fibrosis Cells

With the aim to study the effective anti-inflammatory properties of these new psoralen derivatives, we decided to further characterize the TMA derivatives for their potential activities on the expression of IL-8 gene, known to be involved in the lung inflammatory process in CF. It is firmly established that IL-8 gene expression is regulated at least in part by the NF-*κ*B transcription factor [24–26]; therefore, since molecules inhibiting NF-*κ*B/DNA interactions might exhibit inhibitory activities on NF-*κ*B-regulated genes [27], we were interested in determining the activity of TMA analogues on IL-8 gene expression. More specifically, we were first interested in identifying compounds with low antiproliferative activity, but effective in downmodulating inflammatory gene expression without achieving a full suppression of these genes. We therefore employed IB3-1 cystic fibrosis cells, incubated for 5 h with two concentrations (1 *μ*M and 10 *μ*M) of selected compounds, and then treated with TNF-*α* (100 *μ*g/ml). In the IB3-1 cystic fibrosis cellular model, NF-*κ*B-dependent genes, including the gene encoding for the proinflammatory protein IL-8, generally are activated following infection with *P. aeruginosa* or treatment with TNF-*α* or IL-1*β* [26, 28, 31]. This feature is very important in the pathophysiology of CF, since several clinical complications are caused by exacerbation of this inflammatory response. After 1 day of incubation, cellular RNA was isolated for RT-qPCR analysis. All the TMA analogues found to be active in inhibiting NF-*κ*B/DNA interactions were analyzed for their effects on inhibition of TNF-*α*-induced accumulation of IL-8 mRNA.

The results of RT-qPCR analyses obtained are summarized in Table 3. Our results demonstrated that treatment of IB3-1 cells with TNF-*α* induces a fast and sharp IL-8 gene overexpression. This effect is clearly inhibited by the presence of some TMA derivatives, the most active being **1**, **8**, **26**, **32**, **33**, **34**, **36**, and **38**.

### 3.4. Bioplex Analysis

**Figure 5:**
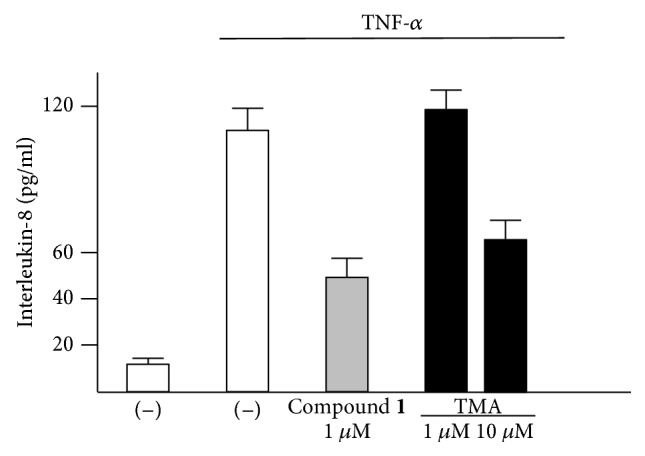
Bioplex analysis of IL-8 release (pg/ml) in IB3-1 cells induced with TNF-*α* in the absence and in the presence of compound **1 (**1 *μ*M); the effects of 1 *μ*M and 10 *μ*M TMA were included for comparison (*n* = 3). IB3-1 cells were treated with compound **1** for 5 hours and then stimulated with TNF-*α* for further 24 hours. The supernatants were analyzed using a Bioplex cytokine assay.

In order to verify the effects on the secretion of IL-8 by IB3-1 cells, Luminex technology was employed to detect and quantify proteins secreted into the medium. This proof-of-principle was conducted on compound **1** since using high concentrations of this compound, no antiproliferative activity was observed (IC_50_ > 800 *μ*M), despite the fact that this compound was one of the most active in inhibiting NF-*κ*B/DNA interactions (see Figure 3). The found results were in full agreement with those obtained studying IL-8 mRNA accumulation, showing that the inhibition of IL-8 is higher than that induced by TMA. This is shown in Figure 5, which supports the decrease of IL-8 expression during treatment with derivative **1** (1 *μ*M), showing about 50% reduction of IL-8 secretion when compared to untreated, TNF-*α*-stimulated cells. This effect was higher than 1 *μ*M TMA and similar to that found with 10 *μ*M TMA. The higher efficiency of compound 1 with respect to TMA was reproducibly observed in three independent experiments. As far as the other cytokines, chemokines, and growth factors that can be analyzed with the Bioplex approach, only minor (less than 10% variation) effects were found for all the secreted proteins analyzed with the exception of the TNF-*α*-inducible IP-10, which was further increased (more than twofold in three independent experiments). Altogether, these data suggest that compound **1** is a very interesting inhibitor of proinflammatory cytokine's activities.

## 4. Discussion

Psoralens are well-known furocoumarins belonging to the class of photosensitizers used for their activity in the treatment of various chronic inflammatory skin diseases, and they are characterized by a differently substituted tricyclic aromatic skeleton, derived from condensation of a coumarin nucleus with a furan ring. Among psoralen-related compounds, the angular angelicin- (ANG-) like isomers are both synthetic and natural compounds that we demonstrated exhibiting interesting pharmacological activities compared with linear psoralens [11].

Recently, we established that 4,6,4′-trimethylangelicin (TMA) is a strong inhibitor of the expression of the IL-8 gene in bronchial epithelial cells in which the inflammatory response has been challenged with *P. aeruginosa* [12], the most common bacterium found in the airways of patients affected by cystic fibrosis (CF). These findings suggested us to continue our research analyzing 38 new analogues of TMA in order to evaluate their biological activities on human bronchial epithelial cells IB3-1, derived from a CF patient with a ΔF-508/W1282X mutant genotype. This cellular system is very attractive, since it is well known that the hallmark in CF airway pathology is a characteristic elevated concentration of proinflammatory cytokines and chemokines, the most important of which seems to be IL-8.

In the field of research about CF, the study of novel and innovative drugs for the treatment of this pathology is constantly evolving in order to ameliorate the clinical conditions of patients. In the conventional treatment of CF, the commonly utilized drugs are nonsteroidal anti-inflammatory drugs (NSAIDs) and steroid derivatives that possess, in addition to great benefits, many known side effects. The search for modern therapies to counteract the inflammation in CF patients is aimed at finding new potential anti-inflammatory drugs with alternative mechanisms of action that may replace the use of typical drugs.

In inflammatory processes that involve patients with CF, NF-*κ*B transcription factor plays a crucial role. Indeed, the expression of many genes encoding for cytokines, chemokines, adhesion molecules, and other proteins involved in inflammation is regulated by NF-*κ*B. The NF-*κ*B dimers, as the predominant p50/p65 heterodimer, following a cascade of intracellular biochemical events activated by extracellular stimuli including cytokines as TNF-alpha (TNF-*α*), are left free to move into the nucleus, where they activate specific proinflammatory genes. For this reason, it is extremely interesting to find new potential anti-inflammatory drugs, which inhibit the action of NF-*κ*B and the subsequent production of cytokines (particularly IL-8).

Because our previous studies demonstrated that TMA inhibits NF-*κ*B/DNA interactions *in vitro* in EMSA experiments at 100 *μ*M concentration [12], band shift analyses were performed to find new derivatives able to inhibit the interaction between purified p50 subunit of NF-*κ*B and DNA-specific target mimicking IL-8 promoter. Considering these preliminary results, new TMA derivatives able to inhibit NF-*κ*B/DNA interaction at lower concentration than *lead compound* TMA were selected to investigate their biological activity on IB3-1 cells induced with TNF-*α*. In this system, NF-*κ*B-dependent genes, including those encoding for the proinflammatory protein IL-8, were investigated. TNF-*α*-induced expression of IL-8 was evaluated by quantitative reverse transcription and polymerase chain reaction (RT-qPCR) in IB3-1 cells incubated with 1 *μ*M and 10 *μ*M of active compounds. All the obtained results are summarized in Table 3.

The most interesting compounds showing relevant anti-inflammatory effects are compounds **1**, **32**, **33**, **34**, and **36** derivatives. As expected by EMSA results, these proralens were able to reduce IL-8 mRNA expression of 57% (1 *μ*M), 73% (1 *μ*M), 66% (1 *μ*M), 68% (1 *μ*M), and 77% (1 *μ*M), respectively, without antiproliferative effects at the used concentrations (IC_50_ > 800 mM). Biological activities of all the analyzed TMA derivatives were observed at low concentrations (1–10 *μ*M), without changes in cell proliferation. Other TMA analogues (**8**, **26**, **33**, and **38**) showed high inhibitory effects (52–83%) on IL-8 expression in IB3-1 cell model, demonstrating their potential power in a possible anti-inflammatory therapy, together with **26**, **38**, **36**, and **32**.

Some analogues, finally, including **2**, **5**, **7**, and **28**, showed similar activity (33–38% of inhibition of IL-8 expression) to the lead compound TMA, even though they were considered not promising in EMSA assay.

The biological data allow to determine a very preliminary SAR (structure-activity relationship). Almost all the active compounds possess a tricyclic backbone with furocoumarin structures. The heteroatoms in the policyclic skeleton must be oxygen atoms, since all isosteric substitutions led to poorly active or inactive compounds (“thio series” **14**–**18**, “FQ series” **17**–**19**, and “triazole series” **31**).

The steric hindrance of the substituent in the furocoumarin skeleton appears detrimental for the activity, since almost all the active compounds are methyl derivatives. Monohydroxymethyl derivatives or monoaminomethyl compounds are still active (**8**, **26**, and **38**), but more hindered compounds are inactive.

The furan ring can be condensed at the 5,6 positions of the central benzene ring (“allo series” **32**–**34**) as well as at the 7,8 positions (**1**, **5**, **8**, **26**, and **28**), without impairing the activity, but not at both the positions at the same time (“difuro series” **29**-**30**). The furan ring can be replaced by a pyran one maintaining a good inhibition of IL-8 expression (“pyran series” **35**-**36**). Moreover, the furan ring must be fully aromatic, since the hydrogenated derivatives are inactive (“dihydro series” **20**–**22**).

The coumarin moiety must be in the *α*-pyron conformation since other structural disposition (*γ*-pyron) affords inactive product (“chromone series” **27**).

Finally, the tricyclic derivatives appear to be the more promising ones: the bicyclic derivatives are active only if a substituent mimicking the furan shape is present at the 8 positions (“coumarin series” **38**).

## 5. Conclusions

Our data conclusively allowed the identification of novel TMA analogues exhibiting improved inhibitory activity on NF-*κ*B/DNA interactions, IL-8 gene expression with only minor effects on cell growth. Although epithelial cells lying the surface of the airway tract are considered good sensors of the activity of promising anti-inflammatory agents, the effect of the most interesting TMA derivatives should be in the future extended also to the whole lung tissues of mice infected *in vivo*, in order to verify the possibility of multiple anti-inflammatory effects on different cells orchestrating the innate immune response in the lung.

## Figures and Tables

**Table 1:** Chemical structure of *lead compound* 4,6,4′-trimethylangelicin (TMA) and of TMA analogues.

Series	Compound
	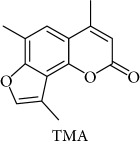
4,6,4′	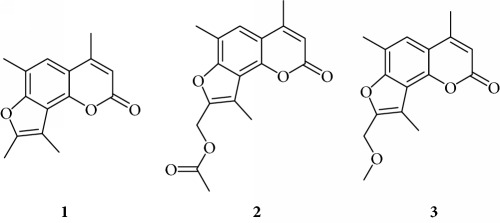
4,6	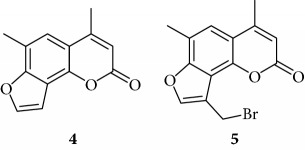
6,4′	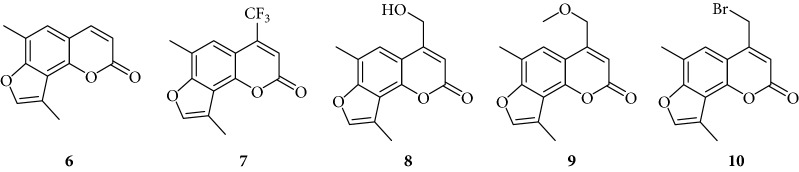
4,4′	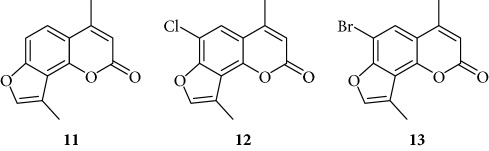
Thio	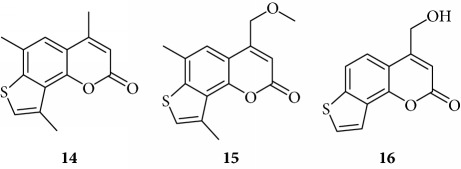
Furoquinolinones (FQ)	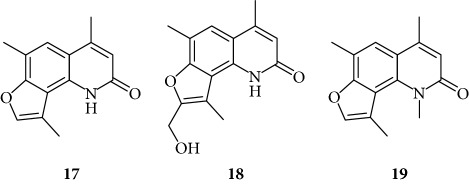
Dihydro	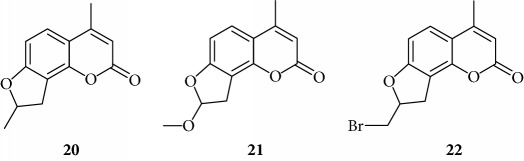
Other angelicins	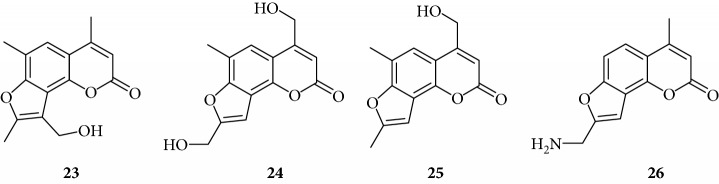
Chromone	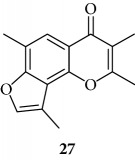
3,4	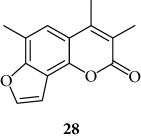
Difuro	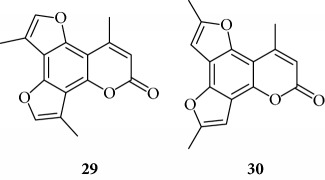
Triazole	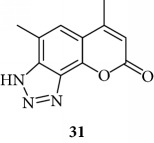
Allo	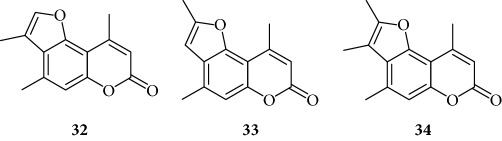
Pyran	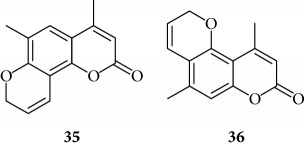
Coumarin	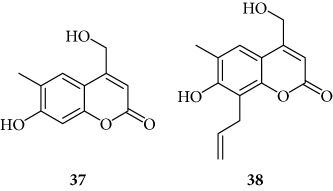

**Table 2:** IC_50_ values calculated after 48 h from treatment of IB3-1 cells with serial dilutions of TMA derivatives.

Derivative	IC_50_ (48 h)
**1**	>800 *μ*M
**2**	>800 *μ*M
**3**	>800 *μ*M
**4**	>800 *μ*M
**5**	485.89 *μ*M
**6**	454.35 *μ*M
**7**	>800 *μ*M
**8**	397.87 *μ*M
**9**	>800 *μ*M
**10**	<50 *μ*M
**11**	181.78 *μ*M
**12**	>800 *μ*M
**13**	>800 *μ*M
**14**	>800 *μ*M
**15**	>800 *μ*M
**16**	>800 *μ*M
**17**	193.07 *μ*M
**18**	>800 *μ*M
**19**	131.12 *μ*M
**20**	154.43 *μ*M
**21**	677.87 *μ*M
**22**	179.92 *μ*M
**23**	451.66 *μ*M
**24**	196.53 *μ*M
**25**	551.23 *μ*M
**26**	90.84 *μ*M
**27**	>800 *μ*M
**28**	>800 *μ*M
**29**	>800 *μ*M
**30**	>800 *μ*M
**31**	>800 *μ*M
**32**	>800 *μ*M
**33**	>800 *μ*M
**34**	>800 *μ*M
**35**	137.31 *μ*M
**36**	>800 *μ*M
**37**	>800 *μ*M
**38**	505.81 *μ*M

**Table 3:** Results of RT-qPCR analysis reporting the % inhibition of IL-8 mRNA in TNF-*α*-induced IB3-1 cells cultured with 1 *μ*M and 10 *μ*M concentrations of TMA derivatives.

Derivative	1 *μ*M (% inhibition)	10 *μ*M (% inhibition)	Inhibition of NF-*κ*B/DNA complex (MIC) *μ*M
**1**	57	56	<12.5 (^∗^)
**5**	33	31	12.5–25
**8**	51	52	25–50
**12**	0	na	12.5
**16**	18	21	25–50
**23**	3	11	2–505
**25**	na	na	50–100
**26**	55	83	50–100
**27**	16	na	<12.5 (^∗^)
**28**	21	36	12.5–25
**29**	na	12	<12.5 (^∗^)
**30**	10	na	12.5–25
**31**	na	na	12.5
**32**	73	52	<12.5 (^∗^)
**33**	68	67	12.5–25
**34**	66	47	<12.5 (^∗^)
**35**	56	37	25–50
**36**	77	74	12.5–25
**37**	na	na	12.5–25
**38**	36	81	25

na: not active at the used concentrations; MIC: minimal inhibitory concentration. ^∗^In the EMSA experiments, 12.5 *μ*M was the minor concentration used.
